# Convention of Medical Teachers, Assembled at Louisville, May 2d, 1859

**Published:** 1859-06

**Authors:** 


### EXTRACTS.

### CONVENTION OF MEDICAL TEACHERS,

### ASSEMBLED AT LOUISVILLE, MAY 2, 1859.

The Convention of Medical Teachers was called under the following resolution, adopted at the eleventh annual meeting of the American Medical Association, held at Washington City, last year:

*Resolved*, That we recommend to all the medical colleges entitled to a representation in this body, that they appoint delegates especially instructed to represent them in a meeting

---

would indicate that there is probably in the blood something intermediate, between the oxygen and the carbonic acid.

to be held at Louisville on Monday, the day immediately preceding the convention of the American Medical Association for the year 1859, at ten o'clock in the morning, at such place as the committee of arrangement shall designate.

At the hour of 10 the convention was called to order, and Prof. Dixi Crosby, of Dartmouth College, Hanover, N. H., was selected as chairman, and Prof. George C. Blackman, of Ohio Medical College, at Cincinnati, as secretary. Prof. Crosby, on assuming the chair, said that, like all his predecessors called upon to preside over deliberative bodies, he had been taken wholly by surprise, and should have declined had not Dr. Frost, of S. C., and Dr. Davis, previously excused themselves from serving. He could bring the convention no qualification for the position, except an earnest desire to serve them; but this and the support of the members, he hoped, would enable him to meet their approval, and conduct the important deliberations satisfactorily.

Rev. J. H. Haywood was then introduced, and invoked the Divine supervision over the proceedings of the body in an earnest and eloquent prayer.

Some discussion then ensued as to the mode of organization, some wishing all medical professors present to act as delegates, and others desiring that each college should have a unit representation. The following resolution was submitted by Dr. David F. Wright, of Shelby Medical College:

*Resolved*, That all members of the Faculties of Medical Colleges now present shall be considered members of this convention, but that where more than one belong to the same college, one of them alone shall vote in behalf of that institution.

After some further interchange of views—all tending to the same wish of full representation—on motion of Dr. A. H. Baker, of Cincinnati, the following substitute was offered and adopted:

*Resolved*, That a committee of three on credentials be appointed by the chair.

Under this resolution, Prof. Crosby selected Drs. Baker, Shattuck and Haskins, as the Committee on Credentials, and the convention took half an hour's recess for the registration of names of delegates.

The Convention was then permanently organized by the reelection of the temporary officers, Prof. Crosby humorously remarking that the delegates were fortunate in this action, inasmuch as they would have no further speech in reference to the honor conferred, etc. He said, that until the Convention should adopt rules for its government, he should limit speeches to ten minutes, and allow no one to speak more that twice on the same subject, without permission.

Dr. Wright's resolution that members from Medical Colleges who are now present, be permitted to take part in debates, but that each college have but one vote, which was again taken up, considered and passed.

Dr. N. S. Davis offered the following, which was adopted:

*Resolved*, That a business committee of five be appointed by the chair, to report propositions for the action of the Convention.

The chair appointed Drs. N. S. Davis, Gunn, Frost, Shattuck and Yandell. After a short recess, to enable this committee to report, they submitted the following through Dr. Davis, the Chairman:

1. *Resolved*, That this convention recognizes the great advantages to be derived from the action of the American Medical Association in prescribing the terms and conditions on which medical degrees shall be conferred, and licences to practice medicine shall be granted; and that an expression of opinion as to methods or periods of instruction from the American Medical Association, should be received with deference and respect, and that all pains should be taken to enforce any rules and regulations recommended by that body.

2. That this convention earnestly recommend the American Medical Association to adopt such measures as will secure the efficient practical enforcement of the standard of preliminary education, adopted at its organization in May, 1847; and that the medical colleges will cheerfully receive and record the certificates alluded to in said standard, whenever the profession generally and the preceptors will see that students are properly supplied with them.

3. That no medical college should allow any term of practice to be a substitute for one course of lectures in the requisitions for graduation.

4. *Resolved*, That Hospital Clinical Instruction constitutes a necessary part of medical education, and that every candidate for the degree of Doctor of Medicine should be required to have attended such instruction regularly for a period of not less than five months, during the last year of his period of medical pupilage.

5. *Resolved*, That every medical college should rigidly enforce the rule requiring three full years of medical study before graduation, and that the diploma of no medical college shall be recognized, which is known to violate this rule.

Prof. Wright, of Nashville, moved that the resolutions of the report be considered *seriatim*—and, the first being taken up, he spoke at length in opposition to it, giving a history of the previous difficulties between the American Medical Association and the medical colleges. He could neither vote for such a resolution; nor could he take any future part in the proceedings of a convention which should adopt it.

Prof. Brainard, of Chicago, thought this convention was asked to take a step fraught with peril to the harmony of the profession and its best interests; it should be met on the threshold, and a solemn protest entered against it. This body did not represent the medical colleges of the country with unanimity—New York, Philadelphia and New Orleans are not represented here, and he must consider their absence as a protest against the assumption of any power on the part of this body or the American Medical Association, to dictate the terms on which the colleges should confer their degrees or receive their students.

The admission of such a resolution would produce hostile factions, both in the profession and in the colleges, and could never receive the sanction of those who had independent, chartered rights to fall back upon. He was opposed to no true improvement in the medical profession, but he did object to shutting that door upon young men desirous of entering the profession, through which we ourselves all had entered.

Without definite action on the resolution, the convention adjourned until 3 o'clock P. M.

AFTERNOON SESSION.

When the convention reassembled, Dr. Bayless offered the following amendments to the first resolution:

1. To substitute in the third line the word "recommending" for "prescribing."

2. To strike out all after the words "deference and respect."

A long discussion ensued on the resolution, which was participated in by Drs. Bayless, Yandell, Palmer, McDowell, Davis, Brainard, Shattuck, Baker and Wright. The differences of opinions seemed almost as various as the number of speeches, and the convention was tying itself into an apparently inextricable entanglement, when an Alexander sprung up, in the person of Prof. L. L. Joynes, of the Medical College of Richmond, Va., who offered the following preamble and resolutions as a substitute for the resolutions from the business committee:

WHEREAS, It appears that a large proportion of the medical colleges of the United States are unrepresented in this convention, and no changes in the present system of education can be effectual unless adopted by the schools generally—

*Resolved*, That it is inexpedient at this time to take any action upon the proposition contained in the report presented by the Special Committee on Medical Education, at the last meeting of the American Medical Association.

*Resolved*, That with the view of obtaining a more general union in counsel and in action, upon this important subject, this convention do now adjourn, to meet again on the day preceding the next annual meeting of the American Medical Association, at the place which may be agreed upon for said meeting, and that the several medical colleges in the United States, be requested to appoint each one delegate to such adjourned meeting of this convention.

These resolutions were amended, at the suggestion of Dr. Wright, to include the appointment of a committee of five, to take into consideration, during the recess, the various matters referred to in the resolutions, and to report thereon at the adjourned meeting.

The vote was demanded on this by colleges, and resulted as follows:

Yeas—Shelby Medical College, Missouri Medical College, St. Louis Medical College, Oglethorpe Medical College, Ohio Medical College, Western Reserve Medical College, Kentucky School of Medicine, Medical College, Richmond, Atlanta Medical College, Rush Medical College—10.

Nays—Medical College, S. C., Medical College, Ga., Medical Department University, Mich., University of Louisville, Cincinnati College of Medicine, Lind University, Iowa University, Medical College, Memphis, Harvard University—9.

The substitute was declared adopted, yeas 10, nays 9, and so the convention stood adjourned until the day preceding the next annual meeting of the American Medical Association.

The chairman appointed the following committee under the above resolution: Drs. Yandell, Shattuck, Blackman, Campbell and Gunn.

